# Nutrient-responsive regulation determines biodiversity in a colicin-mediated bacterial community

**DOI:** 10.1186/s12915-014-0068-2

**Published:** 2014-08-27

**Authors:** Felix JH Hol, Mathias J Voges, Cees Dekker, Juan E Keymer

**Affiliations:** Department of Bionanoscience, Kavli Institute of Nanoscience, Delft University of Technology, Lorentzweg 1, CJ Delft, 2628 The Netherlands; Instituto de Ecología y Biodiversidad, Casilla 653, Santiago, Chile

**Keywords:** Interference competition, Community dynamics, Colicin, Biodiversity

## Abstract

**Background:**

Antagonistic interactions mediated by antibiotics are strong drivers of bacterial community dynamics which shape biodiversity. Colicin production by *Escherichia coli* is such an interaction that governs intraspecific competition and is involved in promoting biodiversity. It is unknown how environmental cues affect regulation of the colicin operon and thus influence antibiotic-mediated community dynamics.

**Results:**

Here, we investigate the community dynamics of colicin-producing, -sensitive, and -resistant/non-producer *E. coli* strains that colonize a microfabricated spatially-structured habitat. Nutrients are found to strongly influence community dynamics: when growing on amino acids and peptides, colicin-mediated competition is intense and the three strains do not coexist unless spatially separated at large scales (millimeters). Surprisingly, when growing on sugars, colicin-mediated competition is minimal and the three strains coexist at the micrometer scale. Carbon storage regulator A (CsrA) is found to play a key role in translating the type of nutrients into the observed community dynamics by controlling colicin release. We demonstrate that by mitigating lysis, CsrA shapes the community dynamics and determines whether the three strains coexist. Indeed, a mutant producer that is unable to suppress colicin release, causes the collapse of biodiversity in media that would otherwise support co-localized growth of the three strains.

**Conclusions:**

Our results show how the environmental regulation of an antagonistic trait shapes community dynamics. We demonstrate that nutrient-responsive regulation of colicin release by CsrA, determines whether colicin producer, resistant non-producer, and sensitive strains coexist at small spatial scales, or whether the sensitive strain is eradicated. This study highlights how molecular-level regulatory mechanisms that govern interference competition give rise to community-level biodiversity patterns.

**Electronic supplementary material:**

The online version of this article (doi:10.1186/s12915-014-0068-2) contains supplementary material, which is available to authorized users.

## Background

Competition within and between species shapes populations, governs community dynamics, and determines biodiversity [1-3]. Competition occurs in two forms: individuals compete indirectly by consuming resources and thus depriving others of those (exploitative competition); and can compete directly by harming others through antagonistic interactions (interference competition) [4]. Direct antagonistic interactions through the production and release of antibiotics have been recognized as key drivers of microbial community dynamics [3,5,6]. Antibiotic-mediated competition is ubiquitous among bacteria and has various eco-evolutionary roles: antibiotics can kill competitors to enable the colonization of crowded habitats [7–9], enhance virulence [10], and facilitate infection [11]. In a broader ecological context, antibiotic-mediated competition has been suggested to drive diversification [6,12,13] and promote biodiversity [6,14–16]. It is becoming increasingly clear that stress responses to DNA damage or nutrient limitation are major inducers of antibiotic production in many species [17]. However, it remains unclear how the regulation of antibiotic-mediated antagonistic traits shapes competition in bacterial communities. We therefore lack understanding of how environmental cues, through such regulatory processes, influence the biodiversity of bacterial communities.

We investigate colicin-mediated chemical warfare by *Escherichia coli* in spatially structured habitats. *E. coli* actively compete with fellow members of the same (and closely related) species by producing narrow-spectrum proteinaceous antibiotics—colicins—and releasing those into the environment [18]. Colicinogeny is widespread in natural *E. coli* populations: approximately half of all naturally occurring *E. coli* is capable of producing at least one type of colicin, while more than half is resistant to at least one colicin, a small fraction of naturally occurring *E. coli* is sensitive to all existing colicins [19–22]. Despite the killing potential that colicin production provides, colicin producer strains often coexist with sensitive and resistant non-producing strains in natural communities [21,22]. This apparent paradox is often explained by means of a cyclical competitive hierarchy—akin to the game of rock-paper-scissors—that may arise from the interaction of a colicin-producer, a resistant non-producer, and a colicin-sensitive strain [3,9,14,18]. Theoretical studies indicate that in a spatially structured habitat, a cyclical competitive hierarchy can stabilize community dynamics and thus support biodiversity [23–25]. In a laboratory implementation of such a system, an *E. coli* strain producing colicin E2 (having DNase activity), a resistant non-producer strain, and a strain sensitive to colicin E2 have been shown to coexist in a dynamic equilibrium: on solid agar plates colicin producers killed and displaced sensitive cells, sensitive cells outgrew and displaced resistant cells that in turn displaced producers. By contrast, in a well-mixed flask lacking spatial structure, biodiversity was rapidly lost because of eradication of the sensitive strain and subsequent outcompetition of the producer by the resistant strain [14].

Coexistence (i.e. the long-term sympatric persistence of types [26]), however, critically depends on the relative competitiveness of the three strains and is sensitive to factors as community size, habitat structure, killing range of the producer, and expression of the colicin operon [27–30]. Additional file 1: Figure S1 presents a spatially explicit model that illustrates the community dynamics for various interaction scenarios. Our model shows that asymmetric competition (e.g. the competitive advantage of producer versus sensitive is larger than resistant versus producer) indeed jeopardizes biodiversity and often leads to the extinction of two strains. As experimental studies indicate that strong asymmetries indeed exist in the colicin-mediated community [31], it is relevant to ask if rock-paper-scissors mediated coexistence of the three strains is robust, or whether it is confined to a small region of parameter-space. Regulation of the colicin operon is likely to be essential in determining the outcome of colicin-mediated competition, as the conditions under which colicin is produced and released govern the community dynamics. Recent findings suggest a link between nutrient conditions (exploitative competition) and colicin production and release (interference competition): production of colicins 1b, K, and the lysis protein of colicin E7, are controlled by nutrient-responsive regulators [11,32,33]. Environmental factors such as the type and amount of nutrients present in a habitat, may thus have a marked impact on colicin-mediated community dynamics and the persistence of biodiversity.

Here, we study the community dynamics of colicin E2 producing, sensitive, and resistant non-producing strains in spatially structured habitats. Using microfabrication, we create microhabitats that mimic the micro-scale spatial structure of natural bacterial habitats such as soil and the gut [34–36]. We investigate the influence of different growth media on community dynamics and the persistence of biodiversity in those habitats. A fluorescent reporter for colicin production allows us to determine under what conditions colicin is being produced and released. Our results demonstrate that *E. coli* utilizes information regarding nutrient conditions to decide whether or not to autolyse and release colicin into the environment. Colicin release, in turn, affects the community dynamics and translates into either loss or persistence of biodiversity in the habitats. In particular, we reveal the significance of carbon storage regulator A (CsrA) in integrating autolysis rates from nutrient availability by *E. coli*. We furthermore show that the three strains can coexist without interacting according to a rock-paper-scissors game and discover an alternative path to coexistence in which CsrA-mediated lysis repression allows producer, sensitive, and resistant cells to thrive in close proximity. Exploring and manipulating the interplay between the environment, regulation, and community dynamics, allows us to understand how molecular-level regulatory mechanisms that govern interference competition give rise to community-level biodiversity patterns.

## Results

### Community dynamics and the persistence of biodiversity depend on nutrient type

**Figure 1:**
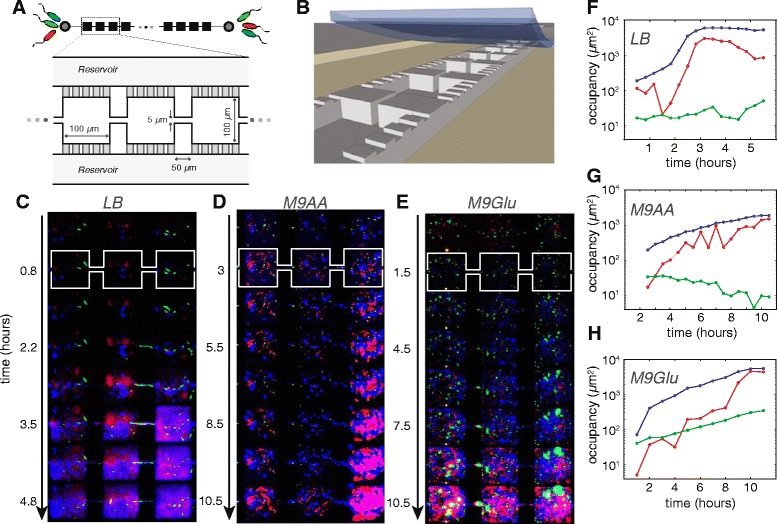
**Community dynamics in spatially structured microhabitats.** **(A,B)** Schematic **(A)** and cartoon **(B)** showing a microhabitat consisting of 85 patches (100 × 100 × 15 *μ*m) connected by corridors (50 × 5 × 15 *μ*m). All 85 patches are connected to growth-medium reservoirs by 180 nm deep slits preventing bacteria from entering the reservoirs but allowing the diffusion of e.g. nutrients and waste. Microhabitats are sealed using a cover slip. **(C-H)** Kymographs (representing space horizontally and time vertically) of representative sections of microhabitat experiments and the corresponding growth curves in LB **(C,F)**, M9-amino acids **(D,G)**, and M9-glucose **(E,H)**. In all three media, producer (shown in blue) and resistant cells (red) grow readily. Sensitive cells (green) show no growth in LB and M9-amino acids, and sometimes turn filamentous (visible in LB in the connector between the center and rightmost patch). In M9-glucose however, sensitive cells show exponential growth (green line in **(H)**) in close proximity to producer and resistant cells.

We investigated the community dynamics of a colicin E2 producer, a resistant non-producer, and a colicin E2 sensitive strain in structured habitats in various growth media. Using microfabrication, we created spatially structured microhabitats consisting of 85 habitat-patches connected by corridors (see Figure 1A and B). Microfabrication allows us to create habitats that are spatially structured at the micrometer scale and perform replica experiments using various growth media [37]. Similar to bacteria living in natural habitats, cells inhabiting a microhabitat can switch between a free-swimming (planktonic) and surface-associated (sessile) lifestyle. Switching between these modes allows cells to develop multicellular aggregates and disperse from those giving rise to a self-structured community that remains dynamic for days [37,38]. Time-lapse microscopy of fluorescently labeled versions of the three strains enables us to study the community dynamics at high spatiotemporal resolution [36] (Additional file 2: Table S1 lists all strains used in this work).

**Figure 2:**
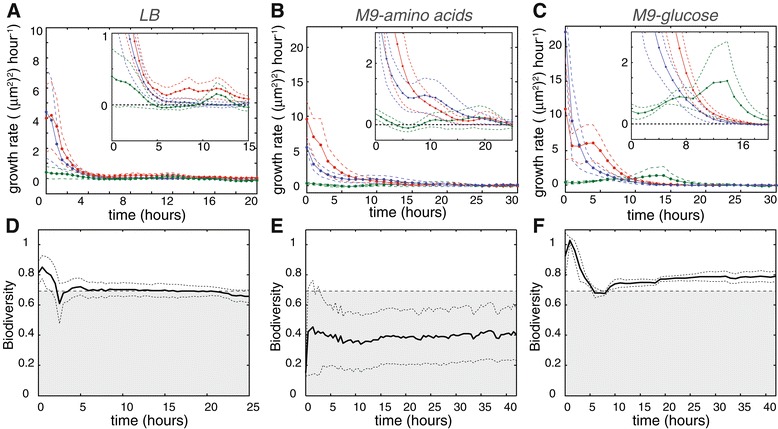
**Growth and biodiversity through time in different media.** **(A-C)** Mean habitat-wide per-capita growth rates through time of producer (blue), resistant (red), and sensitive (green) cells calculated from three replicate microhabitat experiments per medium, the dashed line indicates the standard error of the mean (SEM). Insets show the initial phase in detail and demonstrate that the growth rate of the sensitive strain fluctuates around zero in LB **(A)** and M9-amino acids **(B)** but is positive in M9-glucose **(C)**. **(D-F)** Biodiversity through time is calculated at the level of an entire habitat as Shannon’s index ($H=-\sum _{i=1}^{3} p_{i} \ln p_{i}$, where *p*_*i*_ denotes the frequency of strain *i*), full lines indicate the mean of three independent experiments, dashed lines indicate the SEM. The dashed line at ln(2) depicts maximum diversity for 2 types.

Figure 1C-H shows representative time-sequences and corresponding growth curves of community dynamics in three different media (Lysogeny broth (LB), M9 minimal medium + 1*%* amino acids (M9-amino acids), M9 minimal medium + 0.4*%* glucose (M9-glucose)) where the three strains were inoculated at equal abundance at both ends of the habitat (symmetric inoculation). Interestingly, we find that the different media give rise to distinctly different community dynamics. Producer and resistant cells thrive in all three media, but the fate of the sensitive strain differs dramatically between the media: in both LB and M9-amino acids, sensitive cells show little growth and often turn filamentous or lyse indicating that they are affected by colicin. In M9-glucose medium however, sensitive cells display exponential growth even in close proximity (<20 *μ*m) to growing producer cells. The per capita growth rates of the individual strains calculated from three replica microhabitat experiments per medium (Figure 2A-C), confirm the influence of growth medium on the ability of sensitive cells to colonize a habitat in the presence of producer and resistant cells. In both LB and M9-amino acids, there is some initial growth of sensitive cells which rapidly becomes negligible, and remains zero for the entire duration of the experiment. In M9-glucose on the other hand, the per capita growth rate of the sensitive strain is positive, and sensitive cells even sustain growth after the producer and resistant strains have ceased growth. M9-glucose thus appears to have a remarkable effect on supporting co-localized growth of the three strains and consequently on maintaining biodiversity.

In order to assess the impact of the three growth media on biodiversity quantitatively, we measured the biodiversity of replicate microhabitat experiments by calculating Shannon’s diversity index through time. Shannon’s index reflects the relative abundance of strains in a microhabitat and peaks at a value of ln(3)≈1.1 when all three strains are present at equal frequencies. In both LB and M9-amino acids medium, the biodiversity index stabilizes at values ≤ ln(2), the maximum biodiversity attainable when only 2 strains co-inhabit a habitat (Figure 2D,E). This value reflects the presence of producer and resistant *E. coli* and indicates the (near) extinction of sensitive cells. Often a small number of nondividing (dormant) sensitive cells could be discerned hours after inoculation in LB or M9-amino acids, indicating that sensitive cells are not always entirely eradicated. Since colicin-mediated killing is an active process requiring energy [39], dormant sensitive cells assuming a persister-like state might remain unaffected by colicin as long as they do not resume growth. As evidenced by the per-capita growth rates all three strains show growth in M9-glucose (Figure 2C). This growth translates into a biodiversity index with a value that exceeds ln(2) confirming the long-term coexistence of the three strains (see Figure 2F). Colicin-producing cells do not prevent the growth of colicin sensitive cells in M9-glucose, even when growing in close proximity to each other (i.e. in the same habitat patch). Co-localized growth indicates that coexistence of the three strains in M9-glucose is not the result of spatial rock-paper-scissors dynamics but is caused by other factors, possibly by differential regulation of the colicin operon in different growth media.

To probe whether the community exhibits different dynamics in another spatial setting, we repeated the experiments performed in LB medium by inoculating the producer strain at one end of the habitat, and the resistant and sensitive strains at the opposite end (asymmetric inoculation, three replicate experiments). In this scenario, the two populations—producer only versus sensitive and resistant—enter the habitat ∼13 mm apart and expand towards each other. When asymmetrically inoculated, competition will mainly take place at the colliding population fronts. In contrast, when inoculating all strains from both sides as discussed above, the three strains come face-to-face in all patches and competitive interactions occur between smaller populations over smaller spatial scales. We observed that in the asymmetric scenario, all three strains grow to high densities but the populations do not mix. Instead, an unoccupied zone of several hundred micrometers forms in between the producer population on one side and the resistant/sensitive population on the other (see Additional file 3: Figure S2). This shows that although coexistence of the three strains is not possible in LB at the scale of micrometers (within patches), the three strains may coexist by spatial separation at larger scales (millimeters).

### Colicin induction and release depend on nutrient conditions

The co-localized growth of sensitive and producer cells in M9-glucose prompted us to investigate the nutrient dependence of expression of the colicin E2 operon. Due to producer-sensitive coexistence in M9-glucose, we hypothesized that expression of the colicin operon is likely to be lower in M9-glucose when compared to M9-amino acids and LB medium. Transcription of the colicin E2 operon is part of the SOS regulon and thus strongly repressed by LexA [40]. When the SOS response is induced, RecA stimulates autocleavage of LexA allowing transcription of the colicin operon. The colicin E2 protein is encoded by the first gene in the operon, followed by the immunity gene and the lysis gene (see Additional file 4: Figure S3 for a schematic representation). Premature transcription of the downstream lysis gene is prevented by a transcriptional terminator between the immunity and lysis genes; lysis gene expression thus relies on transcriptional readthrough and is transcribed at a lower rate than colicin E2. SOS responsive genes are generally subject to expression heterogeneity [41] and since colicin is released through self-destructive lysis, it follows that the operon can only be expressed in a fraction of the population to ensure population viability [42].

**Figure 3:**
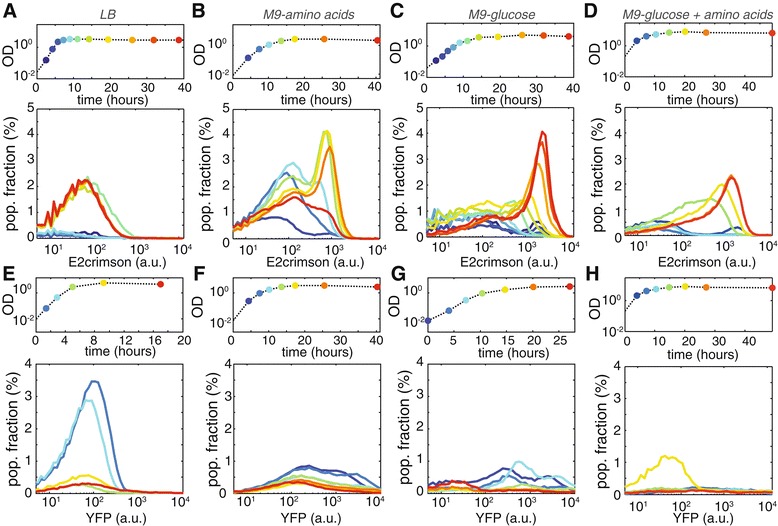
**Colicin production and release depend on growth phase and medium.** **(A-D)** Mean histograms of E2crimson expression measured using flow cytometry at various stages of growth of duplicate experiments in LB **(A)**, M9-amino acids **(B)**, M9-glucose **(C)**, and M9-glucose + amino acids **(D)**. E2crimson serves as a proxy for colicin production. Colors of histograms correspond to the color-coded time-points on the corresponding growth curves (above). The first bin of all histograms (cells not expressing E2crimson) is not included for clarity. The population fraction that expresses E2crimson (i.e. the area under the curve) increases along the growth curve in all media, the expression distribution has a distinct profile for the four media. **(E-H)** YFP expression histograms in LB **(E)**, M9-amino acids **(F)**, M9-glucose **(G)**, and M9-glucose + amino acids **(H)** throughout the growth phase of a strain carrying plasmid pColE2 *Δ* *cel::EYFP*. YFP expression is a proxy for lysis protein production.

We monitored the activity of the colicin E2 promoter (*P*_*sos*_) by placing the fluorescent protein E2crimson under the control of *P*_*sos*_. Figure 3A-D shows E2crimson expression at various phases of growth measured by flow cytometry. As expected, the colicin E2 promoter was only active in a fraction of the producer cells. In agreement with previous studies [43,44], we observed an increase in the population fraction that induced the colicin operon in stationary phase in LB medium (1.6±0.7*%* of cells was induced in exponential phase, versus 26±8*%* in stationary phase; mean of *N*=4 replicate cultures ± SEM). In M9-amino acids, both the fraction of induced cells, and the level of expression increased upon entry into stationary phase and showed a decline in late stationary phase (in exponential phase 17±2*%*, and in late stationary phase 38±4*%* of cells was induced; *N*=3). Interestingly, the expression distribution at the entry of stationary phase in M9-amino acids is trimodal: the majority of cells exhibits no expression, while the cells that do induce the colicin operon can be divided into a low-induction population (likely cells that have just initiated expression) and a high-induction population (cells that have reached the maximum induction level). To our surprise, we found that both the fraction of induced cells and the level of induction of the colicin E2 promoter in M9-glucose were *higher* than in LB and M9-amino acids (in M9-glucose, 22±6*%* and 50±7*%* of cells was induced in exponential and stationary phase, respectively; *N*=4). This indicates that the co-localized growth of producer and sensitive cells observed in microhabitats in M9-glucose (Figures 1 and 2), can not be explained by the absence of colicin production when growing on glucose. Flow cytometry of cultures grown in M9 medium supplemented with glycerol or acetate shows expression profiles comparable to M9-glucose, suggesting that induction of the colicin operon is similar when growing on different sugars (see Additional file 5: Figure S4). The E2crimson expression profile in M9 medium supplemented with both glucose and amino acids (M9-glucose + amino acids, see Figure 3D) is similar to that of M9 supplemented with glucose alone, showing a gradual increase of induction of the colicin operon along the growth curve and reaching a maximum in late stationary phase. The decline of the highly-induced population fraction observed in late stationary phase M9-amino acids cultures, however, does not appear in M9-glucose + amino acids.

To interpret these observations, it is important to realize that the expression distributions in Figure 3A-D represent cells that induced the colicin operon, but did not yet lyse. High induction levels can therefore have multiple causes: they may result from strong induction of the colicin operon, from a reduced lysis rate, or both. In order to distinguish between these scenarios we created a lysis knock-out by replacing the lysis gene (*cel*) with *yfp*. Analyzing the YFP signal of this strain showed that expression of the lysis gene is not directly correlated with activity of the SOS promoter, indicating that additional regulation is at play (see Figure 3E-H). In LB medium, the lysis gene is highly expressed during exponential phase and decreases in activity upon entry into stationary phase. The simultaneous increase in E2crimson signal (i.e. colicin production, Figure 3A) is thus likely to be (partly) caused by E2crimson build-up due to lysis repression in stationary phase. In M9-amino acids, M9-glucose, and M9-glucose + amino acids the lysis gene is expressed at lower levels when compared to LB medium, which may lead to intracellular colicin build-up. In M9-glucose and M9-glucose + amino acids stationary-phase cultures, the high E2crimson signal (reporting colicin expression) and small population fraction expressing YFP (reporting *cel* expression) together suggest that when colicin production is induced in the presence of glucose, lysis is strongly repressed. Although expression of the lysis gene is certainly lower in M9-amino acids when compared to LB, there is significant expression of the lysis gene in late exponential and early stationary phase when cells are cultured in M9 medium supplemented with amino acids only. Expression at those times is strongly repressed when M9-amino acids medium is supplemented with glucose. Together these observations indicate that lysis repression and subsequent colicin build-up are triggered by high-glucose conditions, additional high amino acids content does not reverse this behavior.

**Figure 4:**
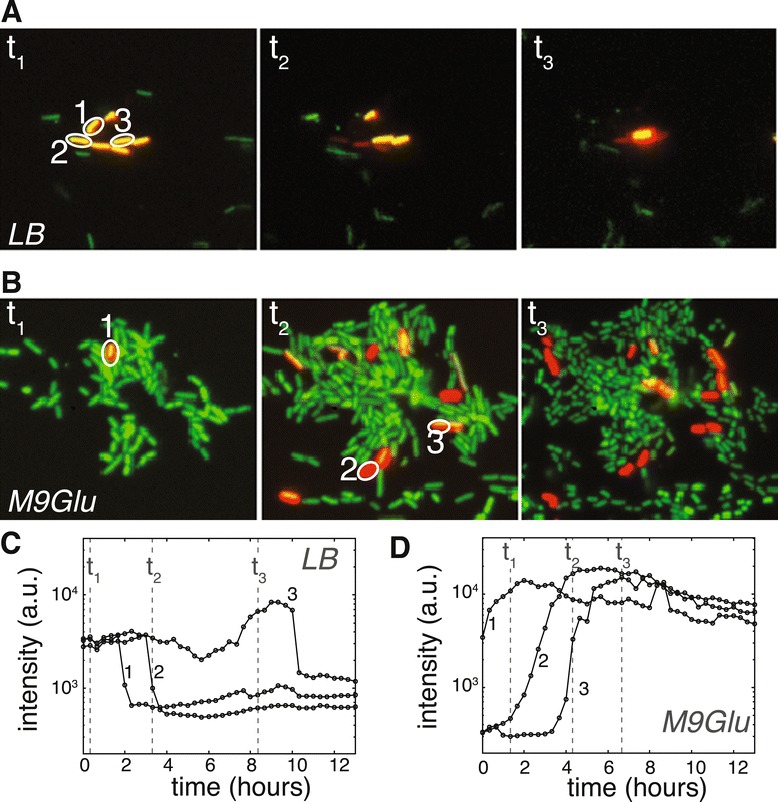
**Colicin release in LB and M9-glucose.** Representative microscopy images and induction-lysis curves of cells growing in LB **(A,C)** and M9-glucose **(B,D)** expressing E2crimson under control of the colicin E2 promoter (shown in red) and having constitutive *yfp* expression (green). Panel **(C)** shows induction-lysis curves of three cells labeled in image *t*_1_ of **(A)**. E2crimson first rises, 2–3 hours after induction cells show a sharp drop in fluorescence indicating a lysis event. A faint red signal shows the remainder of the cell body after lysis. E2crimson time-traces in M9-glucose **(D)** of the cells labelled in **(B)** show that induced cells do not lyse and remain intact for periods longer than 12 hours.

Using time-lapse live-cell imaging, we verified that producer cells indeed show a greatly reduced lysis rate in M9-glucose when compared to LB. Figure 4 presents typical single-cell induction curves in LB and M9-glucose medium, indicating that lysis dynamics vary markedly: in LB medium 44*%* (exponential phase) and 15*%* (stationary phase) of the induced cells (*n*=815) lyse within 5 hours after induction. In M9-glucose however, only 1*%* (irrespective of growth phase) of induced cells (*n*=548) lyse within 24 hours after induction. These observations demonstrate that the vast majority of cells that express the colicin operon in M9-glucose medium do not release the produced colicin into the environment. The co-localized growth of resistant, producer, and sensitive cells in M9-glucose microhabitats is thus likely caused by an inhibition of colicin release and not by the absence of colicin production.

### CsrA determines biodiversity by modulating the lysis rate

**Figure 5:**
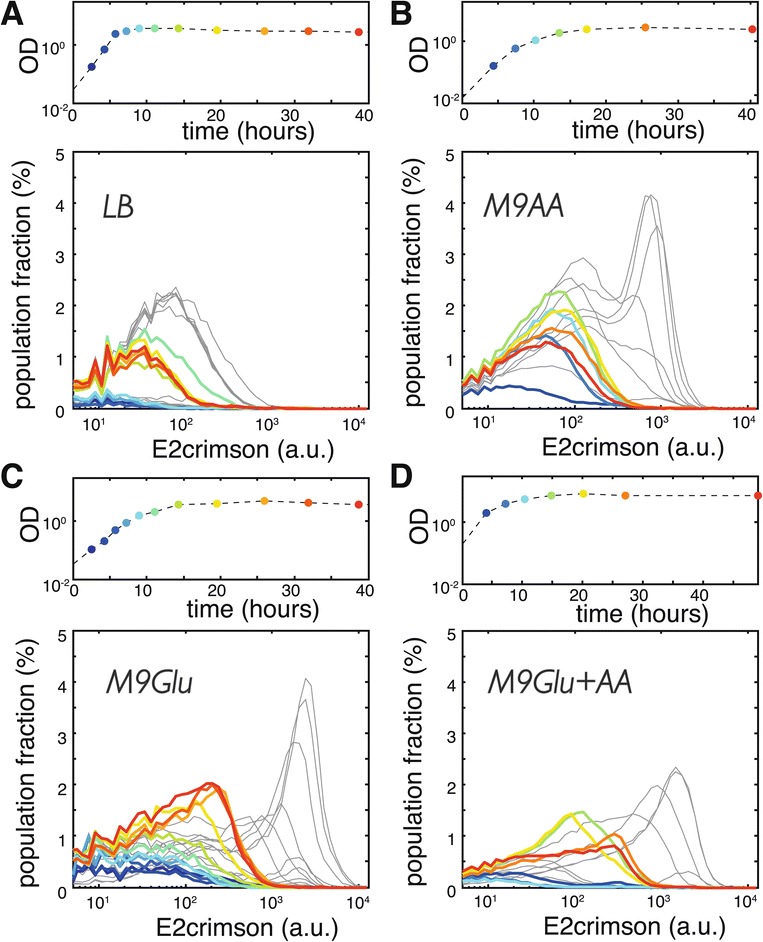
**Relief of CsrA repression prevents colicin build-up through increased lysis.** Expression histograms of E2crimson through the growth phase of producer cells carrying the mutant pColE2-TT plasmid grown in LB **(A)**, M9-amino acids **(B)**, M9-glucose **(C)**, and M9-glucose + amino acids **(D)**, measured using flow cytometry. Histogram colors correspond to time points along the corresponding growth curve (above), grey lines represent histograms of producers carrying the wild-type plasmid (data from Figure 3A-D). Expression profiles of producers harboring the mutant plasmid have shifted to lower expression values and show a decreased fraction of induced cells when compared to expression distributions of producers carrying the wild-type plasmid.

**Figure 6:**
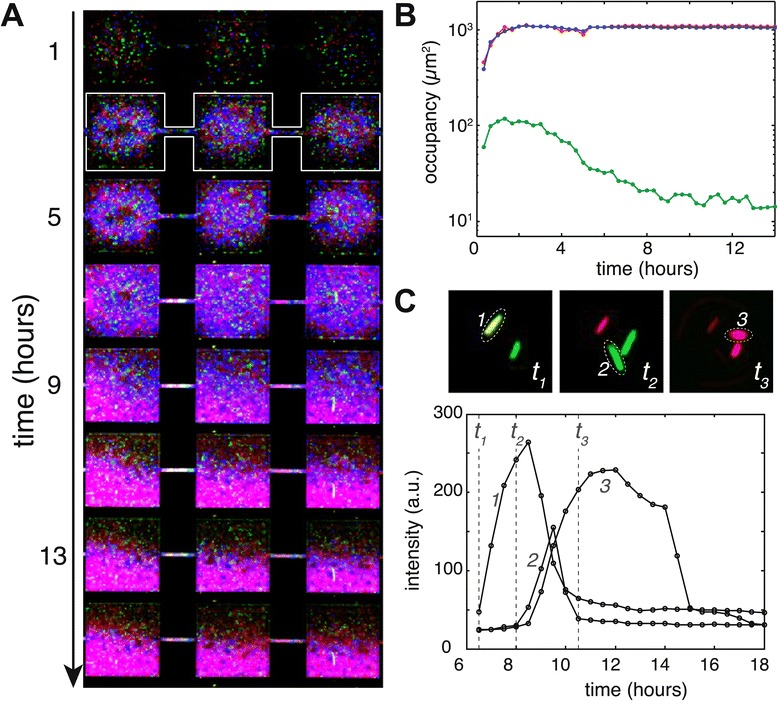
**Relief of CsrA repression increases lysis and results in loss of biodiversity in M9-glucose.** **(A)** Kymograph of a section of a microhabitat showing typical community dynamics of sensitive (green), resistant (red) and pColE2-TT carrying producer cells (blue). Producer and resistant cells grow readily while sensitive cells either show no growth, become filamentous, or die. **(B)** Corresponding growth curves (time versus occupancy) of the section shown in **(A)**. **(C)** Microscopy images and induction-lysis curves of producer cells carrying the pColE2-TT mutant plasmid, the E2crimson reporter for colicin induction (shown in red), and expressing YFP constitutively (green) growing in M9-glucose. Induction-lysis curves of the cells labelled in the microscopy images show a steep drop in fluorescence intensity after induction, indicating lysis.

Next, we investigated the mechanism responsible for the nutrient dependence of the lysis rate. It was recently shown that Carbon storage regulator A (CsrA) can repress translation of the colicin E7 lysis gene by preventing ribosomal binding to its mRNA [33]. Sequencing of the colicin E2 plasmid revealed that the colicin E2 lysis gene also has a CsrA binding site. We therefore hypothesized that the strong reduction of the lysis rate in M9-glucose medium was due to repression by CsrA. Yang *et al.* [33] demonstrated that mutating the first two nucleotides of the CsrA binding site (AC to TT, see Additional file 4: Figure S3) relieves CsrA-mediated repression of the E7 lysis gene. In order to verify that the reduced lysis rate in M9-glucose was indeed caused by CsrA repression, we introduced this mutation into the colicin E2 operon yielding plasmid pColE2-TT. Flow cytometry data of E2crimson expression (our proxy for colicin production) of producer cells harboring pColE2-TT are presented in Figure 5. These data demonstrate that CsrA-mediated lysis-repression indeed has a strong influence on colicin build-up within cells, and that relief of CsrA repression leads to a dramatic change in the expression distribution of the colicin operon. E2crimson expression of producers carrying pColE2-TT has shifted to lower values and is detected in a smaller population-fraction when compared to producers carrying the wild-type colicin E2 plasmid (data indicated in grey in Figure 5). This shift is most prominent in M9-glucose but also clearly visible in LB and M9-amino acids. Using time-lapse live-cell imaging, we verified that producers harboring pColE2-TT indeed readily lyse in M9-glucose (see Figure 6C and Additional file 6: Video S1) which further demonstrates that the shift in the E2crimson expression-distribution of producers carrying the mutant plasmid is due to increased lysis.

The marked effect that CsrA has on producer cell lysis led us to hypothesize that the co-localized growth of sensitive, producer, and resistant cells that we observed in microhabitats filled with M9-glucose medium (Figure 1E and H) may be explained by a reduced lysis rate of the producers. In this scenario, producers do indeed produce colicin in M9-glucose but refrain from releasing it, which safeguards sensitive cells growing in close proximity. In order to address whether this indeed is the case, we investigated the community dynamics of wild-type sensitive and resistant strains, and a producer strain carrying the mutant pColE2-TT plasmid in M9-glucose medium. In three replica microhabitat-experiments, sensitive cells initially showed some growth but ceased growth before resistant and producer cells did (see Figure 6). The growth arrest of sensitive cells in the presence of producers carrying pColE2-TT in M9-glucose, shows that the community dynamics clearly differ from the M9-glucose microhabitat experiments performed with the wild-type producer, where the sensitive strain sustained growth longer than the resistant and producer strains. In agreement with the wild-type community in LB, filamentous growth of sensitive cells could be observed in M9-glucose in the presence of producers carrying pColE2-TT, indicating that producers carrying the mutant plasmid indeed release colicin into the environment and harm sensitive cells. These results demonstrate that CsrA-mediated lysis-repression has a strong impact on the community dynamics of colicin producing, resistant, and sensitive strains and suggest a key role for CsrA in determining whether biodiversity in this community will be maintained or lost.

## Discussion

Tracking the dynamics of colicin producer, resistant non-producer, and sensitive cells at high spatiotemporal resolution has revealed that community dynamics depend critically on the environment. Our results demonstrate that modulation of the colicin release rate determines whether the three strains coexist at small spatial scales, or whether the sensitive strain is eradicated. This observation establishes a direct link between nutrient availability (exploitative competition) and chemical warfare (interference competition). This coupling is implemented by CsrA, which translates environmental cues into antibiotic-mediated community dynamics. CsrA was first discovered as a master regulator of carbon metabolism [45], but is now also emerging as a regulator of virulence genes, toxin secretion, and other behaviors [46–49]. Both carbon metabolism and toxin secretion are important parameters for determining the success of *E. coli* when colonizing its host [50,51]. Our findings place CsrA at the nexus of these processes and it is therefore possible that CsrA is an important factor in determining the colonization success of commensal and pathogenic *E. coli*. Competition between colicin producing and sensitive strains in mouse models has yielded outcomes ranging from eradication of the sensitive strain to long-term coexistence [9,51–54], our results suggest that variations in nutrient conditions may explain these differences.

Chemical warfare through colicin production and release is costly behavior, the strategy adopted by producer cells thus likely reflects how the competitive benefit of chemical warfare compares to its costs. Our experiments show that the chemical warfare strategy adopted by colicin producer cells depends on nutrient conditions: colicin production and release are highest when cells grow exponentially on peptides and amino acids (e.g. in LB medium) whereas colicin release is low in stationary-phase glucose cultures. Both the availability and the type of nutrients thus affect whether producer cells engage in chemical warfare or refrain from it. The nutrient-availability dependence of the chemical warfare strategy can be understood from the perspective of a cost-benefit analysis, however, the adaptive virtue of the nutrient-type dependence is unclear at present. The situation where producer cells produce colicin but do not release it (i.e. stationary-phase glucose cultures) suggests colicin is being stored for later use, possibly as a bet-hedging strategy.

Previous work [14] suggested that at large spatial scales (mm–cm range) for a specific temporal modulation, rock-paper-scissors game dynamics can lead to long-term coexistence of the three strains in LB medium (note that ref. [14] removed all produced colicin from the system and replenished nutrients at daily intervals). Yet, despite its appealing simplicity as a mechanism to maintain biodiversity, stable rock-paper-scissors game dynamics may be confined to a relatively minor region of parameter space [55]. The periodic removal of colicin for instance, effectively increases the cost of production which is to the advantage of the sensitive strain and may be essential for the long-term stability of rock-paper-scissors dynamics. Increasing the cost of production may partially compensate for the asymmetry in interactions (i.e. the competitive advantage of the producer versus sensitive being greater than the advantage of the sensitive versus resistant and resistant versus producer [31]) which may otherwise lead to extinction of the sensitive strain as our experimental and modeling efforts in conjunction with theory [27] suggest. In our system, the three strains do not coexist in LB when co-colonizing a microhabitat. Only when the strains are colonizing the microhabitat from opposite ends, the populations remain spatially separated and coexist at the scale of the entire habitat, not within patches. Theory has suggested that cellular dispersal is a key parameter in determining community dynamics [25], in addition to that, the killing range of colicin—set by its diffusion rate—is likely to be an important parameter setting the spatial scale at which coexistence emerges [55].

## Conclusions

The observation that colicin-producer and sensitive cells show exponential growth in close proximity in M9-glucose, together with our characterization of colicin-operon expression, puts forward an alternative hypothesis explaining coexistence of the three strains. When growing on sugars, the lysis rate of colicin producing cells drops drastically and interference competition is reduced to a minimum, this allows sensitive, resistant and producer cells to coexist at micrometer scales. In contrast, when growing on amino acids and small peptides, producers are more prone to produce and release colicin, and biodiversity is only maintained at larger spatial scales. It is interesting to note that in some natural habitats *E. coli* mainly grows on sugars and sugar acids (i.e. the intestine), whereas amino acids and peptides are the source of growth in other habitats (i.e. the urinary tract) [50,56]. Our results demonstrate that colicin-mediated chemical warfare results in population dynamics with disparate trajectories in these environments. The available nutrients in a habitat thus appear to be an strong driver of interference competition which may determine the success of colonizing strains. Our study highlights the importance of the regulation of antagonistic traits, which, in response to environmental cues, can have a profound influence on biodiversity.

## Methods

### Strains and growth conditions

All strains used in this study are listed in Additional file 2: Table S1 and were derived from the strains used in ref. [14] originally described in ref. [57]. Fragments coding for fluorescent proteins and kanamycin resistance controlled by the lac promoter were integrated into the chromosome, replacing the lacIZ region, using the Quick & Easy *E.coli* Gene Deletion Kit (Gene Bridges). Colicin E2 producer strains were obtained by transforming the fluorescently labelled sensitive strains (BN1051, BN1054, BN1073) with purified pColE2 plasmid isolated from an overnight culture of strain BN1010 (the producer strain used in ref. [14]) using the QIAGEN Mini-Prep kit, to yield strains BN1079, BN0181, and BN1083. The TurboRFP labelled producer strain (BN1173) was directly derived from BN1010 by inserting the TurboRFP fragment into its lacIZ region. TurboRFP and mCherry labelled colicin E2 resistant non-producer strains were obtained by plating an overnight culture of sensitive strains (BN171 and BN1073) on colicin E2 selective plates to obtain strains BN1175 and BN1075 (LB plates containing 100 *μ*L crude colicin E2 obtained and filter sterilized from an overnight culture of producer cells were used for selection). After overnight incubation at 37 °C on colicin E2 selective plates, resistant colonies were selected. CFP and YFP labelled resistant strains (BN1056 and BN1085, respectively) were directly derived from BN1011 by inserting fragments coding for the fluorescent proteins in the lacIZ region. Sequencing of the *btuB* region of all colicin E2-resistant strains revealed the presence of IS*2* elements. It has previously been reported that disruption of the *btuB* receptor confers colicin E2 resistance [58]. M9 broth (Sigma-Aldrich) and Lysogeny broth (LB, Sigma-Aldrich) were prepared according to the manufacturer’s instructions. M9 minimal medium was prepared by adjusting the pH of the M9 broth to 7.4 and adding MgSO _4_ and CaCl _2_ to final concentrations of 2 mM and 100 *μ*M, respectively. To obtain M9-glucose, 0.4% glucose was added to M9 minimal medium; to obtain M9-amino acids, 1% protein hydrolysate amicase (Fluka) was added to M9 minimal medium; to obtain M9-glucose + amino acids, 0.4% glucose and 1% protein hydrolysate amicase was added to M9 minimal medium; 0.4% glycerol was added to M9 minimal medium to obtain M9-glycerol; M9-acetate was prepared by adding 0.55% acetate to M9 minimal medium. All cultures were grown at 37°C and shaken at 200 rpm.

### Design and construction of the colicin-promoter reporter-plasmid pProm-E2

Primers 5’ – GTTTCTTCCGATCGGACATGTCCATGAGTATGTGATATCCGG – 3’ and 5’ – GAAGAAA CCTCGAGCGGCCACCATTAATGTTACC – 3’ were used to amplify the promoter region of pColE2, yielding a PCR product with PciI and XhoI restriction-site overhangs. This included an upstream terminator region, SOS-box, sigma-factor binding site, ribosomal binding site, and the first 29 amino-acids encoding for colicin E2. The purified PCR product was restricted using PciI and XhoI, purified (25 ng/ *μ*L) and ligated into linearized pE2-Crimson (Clontech, linearized by PciI and SalI, 30 ng/ *μ*L) to yield plasmid pProm-E2. Strains BN1010, BN1079, and BN1081 were subsequently transformed by electroporation of the heat-inactivated ligation mixture and plated out onto ampicillin-containing plates for over-night incubation.

### Design and construction of pColE2-TT

The WT plasmid pColE2 was PCR amplified using the mutagenesis primers 5’ – GGCATTCTTTCACATTAAGGAGTCGTTATGAAAAAAATAACAG – 3’ and 5’ – CTGTTATTTTTTTCATAACGACTCCTTAATGTGAAAGAATGCC – 3’. These primers introduced the AC to TT mutation, which releases CsrA repression of the cel transcript [33]. Transformants were selected on Colicin E2-containing LB-agar and mutants were screened using plasmid sequencing.

### Design and construction of pColE2 *Δ*cel::EYFP

In-frame replacement of the *cel* gene by EYFP resulted in a mutant plasmid that retained the wild-type 5’UTR (CsrA binding site) and 12 nucleotides 5’ of the lysis gene, followed by a 6 nucleotide GGATCC “scar”. The pColE2 backbone was amplified using primers 5’ – AAGGATCCTATTTTTTTCATAACGACTCCTTGTTGTG – 3’ and 5’ – AATCTAGACCCGAAATCCTCTTTGACAAAAACAAAGC – 3’, which introduced a BamHI and an XbaI site to the 5’ and 3’ ends of the plasmid backbone, respectively. pEYFP (BD biosciences) was amplified using 5’ – AAGGATCCGTGAGCAAGGGCGAGGAG – 3’ and 5’ – AATCTAGATTACTTGTACAGCTCGTCCATGCCG – 3’. Both PCR fragments were digested using XbaI and BamHI and ligated. TOP 10 *E. coli* (Invitrogen) were transformed with the product of the ligation reaction and mutants were screened by colony PCR and confirmed by sequencing.

### Flow-cytometry

Flow cytometry was performed using a FACScan flow cytometer (Becton Dickinson) controlled using FlowJo software. YFP was excited at 488 nm and emission was filtered using a 530/30 band-pass filter, E2crimson was excited at 561 nm and emission was filtered with a 615/25 band-pass filter. Overnight cultures were diluted to an optical density of 0.01 in 30 mL growth medium in 500 mL erlenmeyer flasks. Cultures were incubated at 37°C and shaken at 200 rpm. At set time intervals 100 *μ*L of culture was taken, diluted in 1 mL growth medium and gently vortexed for 5 seconds to be used for flow cytometry. At least 4×10^4^ cells were counted per sample. Bacteria were identified by forward and side scatter and data was analyzed in a custom made MatLab script.

### Microhabitat experiments

Microhabitats were fabricated in silicon following a previously published protocol [37]. The microhabitats consist of 85 patches (100 × 100 × 15 *μ*m) connected by corridors (50 × 5 × 15 *μ*m) and are connected to reservoirs by 180 nm shallow slits. The slits prevent bacteria from entering the reservoirs but allow the diffusion of e.g. nutrients and waste. The total volume of the reservoirs is ∼15 times larger than the total volume of the bacterial habitat ensuring that enough nutrients are available for long-term experiments. Two ports to inoculate bacteria were drilled through the silicon, one at each end of the habitat. Microhabitats were closed by bonding to polydimethylsiloxane (PDMS) coated cover-slips. Strains were grown overnight (37°C, 200 rpm) and separately diluted 1/100 in fresh medium, all culturing prior to inoculating the microhabitat was done in the same medium as used during the experiment. After growing to mid-log phase, cells were spun down and washed in the appropriate medium to prevent colicin from the producer culture to carry over to the microhabitat. The three strains were mixed at equal frequency (i.e. all strains 1/3) for symmetrically inoculated experiments and 2 *μ*L of the mixture was applied to both inlet ports. Asymmetrically inoculated experiments were performed by inoculating the sensitive and resistant strains at equal frequency in one port and inoculating the producer strain at the opposite port. Before inoculation microhabitats were filled with fresh medium.

### Image acquisition and analysis

Microhabitats were imaged at 15 minute (LB and M9-amino acids) or 30 minute (M9-glucose) intervals using an inverted Olympus IX81 microscope equipped with a 20x (N.A.= 0.75) objective, a Neo sCMOS camera (Andor) and a motorized stage (Marzhauser) controlled using *μ*Manager software [59]. The sample was illuminated using an X-cite 120 Q (Lumen dynamics) light source. The microscope was enclosed in a home-built environmental chamber warmed to 37°C. All experimental data presented in this work were obtained at 37°C, additional microhabitat community-dynamics experiments performed at 26°C showed dynamics similar to experiments performed at 37°C. Images were processed in MatLab using a custom script. Fluorescent proteins do not have the same brightness, and more importantly react in different ways to growth phase and environmental factors like conditioning of the medium and population density. As a result, fluorescent intensity is not a good proxy for measuring biomass. To circumvent the problems associated with intensity measurements, we converted all images to occupancy data using a previously published protocol [38] which measures the area a strain occupies.

## Additional files

Additional file 1
**Figure S1.** Coexistence of cyclically competing species depends critically on interaction symmetry. Using Monte Carlo methods we simulated a spatial stochastic process with four states representing three species and vacancy $\{\mathrm {R}, \mathrm {P}, \mathrm {S}, \varnothing \}$. Here we show time series of lattice configurations of an interacting particle system [60] defined on a two dimensional lattice of 256×256 sites with periodic boundary conditions (torus) and a von Neumann neighborhood. Simulation details are included in the additional file. **(A)** In a fully symmetric scenario, where the replacement rates of the three pairs (*σ*_R_=*σ*_P_=*σ*_S_=1) and the individual mortality rates (*m*_R_=*m*_P_=*m*_S_=0.4) are all equal, the three strains coexist for long times in a dynamic equilibrium. **(B,C)** Equal mortality rates (*m*_R_=*m*_P_=*m*_S_=0.4) combined with asymmetric interaction rates permit the survival of only one species. In **(B)** the interaction rates span three orders of magnitude (*σ*_R_=0.1, *σ*_P_=0.01, *σ*_S_=0.9) and thus are strongly asymmetric leading to a quick eradication of P (green) and eventual dominance of R (red). In **(C)** the interaction rates are only moderately different (*σ*_P_=*σ*_R_=0.1 and *σ*_S_=0.6) and still only a single species prevails. **(D)** When one species has a slightly increased mortality rate (*m*_R_=*m*_P_=0.4, *m*_S_=0.44) and the interaction rates are close to symmetric (*σ*_P_=*σ*_R_=0.1 and *σ*_S_=0.2) also eventually only one species dominates. The interaction scenarios presented above illustrate that rather small asymmetries in interaction- and/or mortality-rates can have a strong impact on community dynamics and the maintenance of biodiversity. In natural systems that exhibit cyclical competitive hierarchies, interaction rates are usually not equal and may even differ by orders of magnitude in the colicin-mediated community [14,31]. Although cyclical competitive hierarchies are likely to have an ecological role in reducing the intensity of competition, the model presented above illustrates that rock-paper- scissors game dynamics may only be a robust mechanism to maintain biodiversity in a relatively minor region of parameter space. Additional file 2
**Table S1.** List of strains used in this work. All strains were derived from the strains used in ref. [14] which were derived from strain BZB1011 originally described in [57]. Additional file 3
**Figure S2.** Asymmetrically inoculated microhabitat. **(A)** Typical kymograph of a microhabitat asymmetrically inoculated with producer cells from the left and a 50/50 mixture of resistant and sensitive cells from the right. Space is depicted horizontally and time vertically, each pixel represents a single habitat patch and is color-coded according to its producer (blue), resistant (red) and sensitive (green) occupancy. Yellow pixels indicate the presence of resistant and sensitive cells, purple pixels represent patches in which both producer and resistant cells are present, the intensity of the colors scales linearly with the area fraction of the patch that is occupied. Both invading populations readily colonize the habitat, but the population fronts do not collide and an unoccupied zone of several 100 *μ*m separating the populations remains. The separation is not caused by an obstruction, as a few resistant cells can be discerned in the left-most patches colonized by producer cells (purple patches on the left side of the kymograph starting from *t*=15 hours, and single red cells in **(B)**). It is also interesting to note that the resistant-sensitive population coming from the right is yellow near the entrance of the habitat but turns red towards the left. This indicates that the population invading from the right is dominated by resistant cells in proximity to the producers and is a mixture of sensitive and resistant cells closer to the entrance. **(B,C)** Zoom-in of the areas indicated with B and C in the kymograph of **(A)**, scale bars indicate 50 *μ*m. Additional file 4
**Figure S3.** Organization of the colicin E2 operon. Colicin E2 is a group A nuclease colicin, group A nuclease colicins all have the same general structure and are under control of a LexA repressed SOS promoter (*P*_*sos*_). *P*_*sos*_ contains two LexA boxes to which two LexA dimers can bind. When the SOS response is induced, RecA stimulates autocleavage of LexA allowing transcription of the operon. Due to a transcriptional terminator (*T*_1_) between the immunity (*cei*) and lysis (*cel*) genes, transcription can result in a full length mRNA and a more abundant shorter mRNA. The full length mRNA corresponds to the entire operon and contains the colicin protein (*cea*), the immunity protein and the lysis protein; the short mRNA corresponds to the colicin and immunity proteins only. In addition to the SOS promoter, the immunity gene also has its own constitutive promoter (*P*_*im*_) which ensures that there is always enough immunity protein present to bind all colicin and prevent it from killing the producing cell itself. The RNA secondary structure of the T1 terminator as predicted by mfold [61] is depicted on the right. CsrA can bind to this structure and prevent translation of the lysis protein by obscuring the Shine-Dalgarno sequence (SD) and preventing ribosomal binding. Mutating the AC nucleotides (shown in red and indicated by arrows) to TT prevents CsrA binding and thus relieves CsrA-mediated lysis repression. Additional file 5
**Figure S4.** Expression of the colicin operon in M9-glycerol and M9-acetate. **(A,B)** Mean histograms of E2crimson expression at various stages during growth of duplicate experiments in M9-glycerol **(A)** and M9-acetate **(B)**. E2crimson is under control of the colicin E2 promoter and thus serves as a proxy for colicin production. Colors of histograms correspond to time points on the growth curves having the same color, the first bin (cells not expressing E2crimson) is not included for clarity. There is a clear growth phase dependence. Expression profiles are similar to cultures grown in M9-glucose. Additional file 6
**Video S1.** Producers carrying pColE2-TT readily lyse in M9-glucose. Video showing producer strain BN1705 (shown in blue) and sensitive strain BN1051 (shown in green) in a microhabitat filled with M9-glucose, two habitat patches (of 100 × 100 × 15 *μ*m) and the corridor connecting them can be seen. The producer carries the mutant colicin plasmid (pColE2-TT) and the colicin-promoter reporter-plasmid pPromE2. Red fluorescence indicates activity of the *P*_*sos*_ colicin promoter. Producers cells that induce the colicin operon turn bright red, many induced producers lyse within 4 hours after induction. Sensitive cells cease growth when colicin is released. Images were acquired at 30 minute intervals.
