# Supplementary material for: Nutrient-responsive regulation determines biodiversity in a colicin-mediated bacterial community
Source: BMC Biol. 2014 Aug 27;12:68. doi: 10.1186/s12915-014-0068-2 (PMC4161892; doi:10.1186/s12915-014-0068-2)
Supplement: Additional file 1 — Figure S1. Coexistence of cyclically competing species depends critically on interaction symmetry. Using Monte Carlo methods we simulated a spatial stochastic process with four states representing three species and vacancy $\{\mathrm {R}, \mathrm {P}, \mathrm {S}, \varnothing \}${R,P,S,∅}. Here we show time series of lattice configurations of an interacting particle system [60] defined on a two dimensional lattice of 256×256 sites with periodic boundary conditions (torus) and a von Neumann neighborhood. Simulation details are included in the additional file. (A) In a fully symmetric scenario, where the replacement rates of the three pairs (σ R=σ P=σ S=1) and the individual mortality rates (m R=m P=m S=0.4) are all equal, the three strains coexist for long times in a dynamic equilibrium. (B,C) Equal mortality rates (m R=m P=m S=0.4) combined with asymmetric interaction rates permit the survival of only one species. In (B) the interaction rates span three orders of magnitude (σ R=0.1, σ P=0.01, σ S=0.9) and thus are strongly asymmetric leading to a quick eradication of P (green) and eventual dominance of R (red). In (C) the interaction rates are only moderately different (σ P=σ R=0.1 and σ S=0.6) and still only a single species prevails. (D) When one species has a slightly increased mortality rate (m R=m P=0.4, m S=0.44) and the interaction rates are close to symmetric (σ P=σ R=0.1 and σ S=0.2) also eventually only one species dominates. The interaction scenarios presented above illustrate that rather small asymmetries in interaction- and/or mortality-rates can have a strong impact on community dynamics and the maintenance of biodiversity. In natural systems that exhibit cyclical competitive hierarchies, interaction rates are us [file 12915_2014_68_MOESM1_ESM.pdf]

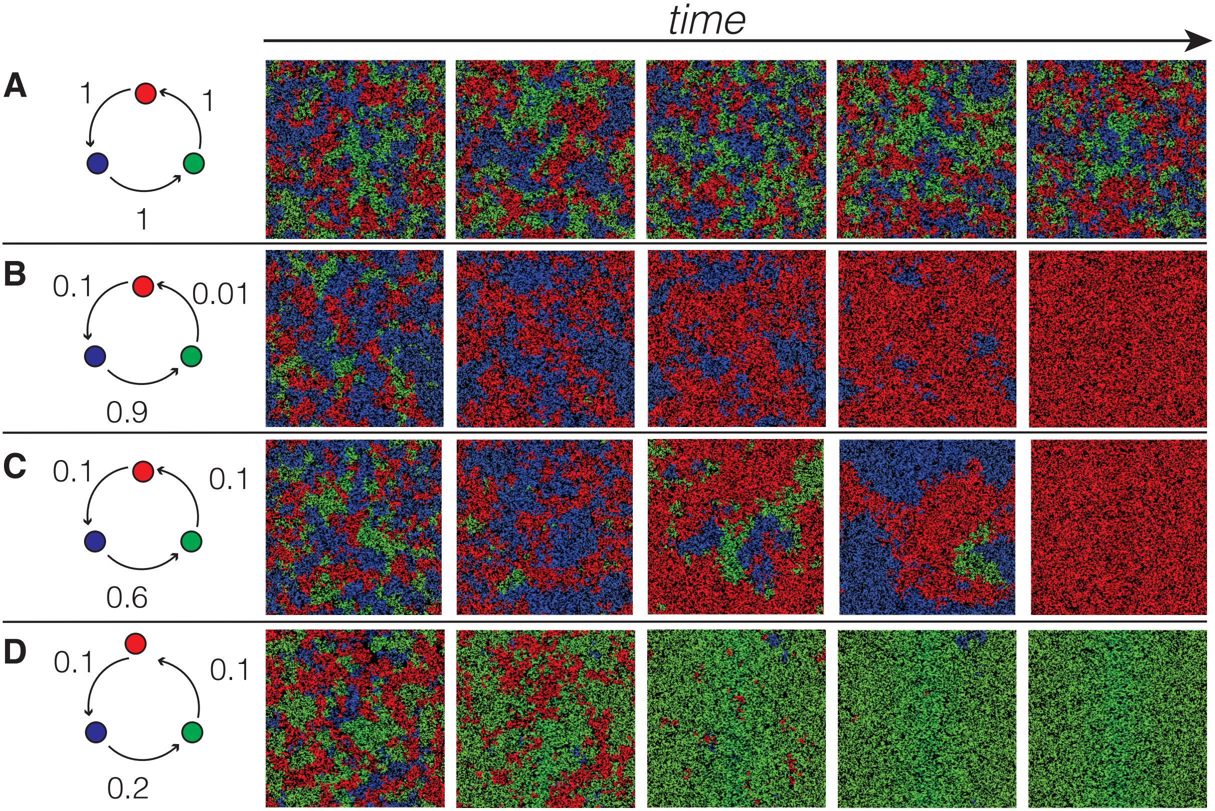

Three contact processes representing each species spread on the lattice with birth rates $\beta_{\mathrm{R,P,S}} = 1$ and die with rates $m_{\mathrm{R,P,S}}$. The three species play a rock-paper-scissor game upon encounters. The simulation proceeds iteratively as follows: pick a random (focal) site (x) and pick a random neighbor (y) out of the four neighbors of the focal site. If both the focal site and the neighbor are vacant, nothing happens; if the focal site is vacant and the neighbor is occupied, the respective neighbor replicates into the focal site with probability one. Thus, $\varnothing_x + \mathrm{R}_y \rightarrow \mathrm{R}_x + \mathrm{R}_y$, $\varnothing_x + \mathrm{P}_y \rightarrow \mathrm{P}_x + \mathrm{P}_y$, and $\varnothing_x + \mathrm{S}_y \rightarrow \mathrm{S}_x + \mathrm{S}_y$. In case the focal site is occupied, it has a probability of dying equal to m_R, m_P, or m_S when occupied by R, P, or S, respectively. Thus, $\mathrm{R}_x \xrightarrow{m_\mathrm{R}} \varnothing_x$, $\mathrm{P}_x \xrightarrow{m_\mathrm{P}} \varnothing_x$, and $\mathrm{S}_x \xrightarrow{m_\mathrm{S}} \varnothing_x$. If the particle does not die, it interacts with a random neighbor following a cyclical competitive hierarchy defined by the rock-paper-scissor game: Rock takes over Scissor with probability σ_R: $\mathrm{S}_x + \mathrm{R}_y \xrightarrow{\sigma_\mathrm{R}} \mathrm{R}_x + \mathrm{R}_y$, Paper takes over Rock with probability σ_P: $\mathrm{R}_x + \mathrm{P}_y \xrightarrow{\sigma_\mathrm{P}} \mathrm{P}_x + \mathrm{P}_y$, and Scissor takes over Paper with probability σ_S: $\mathrm{P}_x + \mathrm{S}_y \xrightarrow{\sigma_\mathrm{S}} \mathrm{S}_x + \mathrm{S}_y$.

(**A**) In a fully symmetric scenario, where the replacement rates of the three pairs ($\sigma_\mathrm{R} = \sigma_\mathrm{P} = \sigma_\mathrm{S} = 1$) and the individual mortality rates ($m_\mathrm{R} = m_\mathrm{P} = m_\mathrm{S} = 0.4$) are equal, the three strains coexist for long times in a dynamic equilibrium. (**B,C**) Equal mortality rates ($m_\mathrm{R} = m_\mathrm{P} = m_\mathrm{S} = 0.4$) combined with asymmetric interaction rates permit the survival of only one species. In (B) the interaction rates span three orders of magnitude ($\sigma_\mathrm{R} = 0.1$, $\sigma_\mathrm{P} = 0.01$, $\sigma_\mathrm{S} = 0.9$) and thus are strongly asymmetric leading to a quick eradication of P (green) and eventual dominance of R (red). In (C) the interaction rates are only moderately different ($\sigma_\mathrm{P} = \sigma_\mathrm{R} = 0.1$ and $\sigma_\mathrm{S} = 0.6$) and still only a single species prevails. (**D**) When one species has a slightly increased mortality rate ($m_\mathrm{R} = m_\mathrm{P} = 0.4$, $m_\mathrm{S} = 0.44$) and the interaction rates are close to symmetric ($\sigma_\mathrm{P} = \sigma_\mathrm{R} = 0.1$ and $\sigma_\mathrm{S} = 0.2$) also eventually only one species dominates.
