# Supplementary material for: Nutrient-responsive regulation determines biodiversity in a colicin-mediated bacterial community
Source: BMC Biol. 2014 Aug 27;12:68. doi: 10.1186/s12915-014-0068-2 (PMC4161892; doi:10.1186/s12915-014-0068-2)
Supplement: Additional file 2 — Table S1. List of strains used in this work. All strains were derived from the strains used in ref. [14] which were derived from strain BZB1011 originally described in [57]. [file 12915_2014_68_MOESM2_ESM.pdf]

Table S1. List of strains used in this work. All strains were derived from the strains used in ref. [14] which were derived from strain BZB1011 originally described in [57].

ID	Plasmid	Characteristics	ColE2-specific phenotype	Reference
**Bacteria**				
BN1009		W3110; *gyrA*	Sensitive	[14]
BN1073		W3110; *gyrA*; Kan^r; Δ*lacIZ*::mCherry	Sensitive	this work
BN1051		W3110; *gyrA*; Kan^r; Δ*lacIZ*::ECFP	Sensitive	this work
BN1054		W3110; *gyrA*; Kan^r; Δ*lacIZ*::EYFP	Sensitive	this work
BN1171		W3110; *gyrA*; Kan^r; Δ*lacIZ*::TurboRFP	Sensitive	this work
BN1011		W3110; *gyrA*; *btuB*::IS*2*	Resistant	[14]
BN1075		W3110; *gyrA*; *btuB*::IS*2*; Kan^r; Δ*lacIZ*::mCherry	Resistant	this work
BN1056		W3110; *gyrA*; *btuB*::IS*2*; Kan^r; Δ*lacIZ*::ECFP	Resistant	this work
BN1085		W3110; *gyrA*; *btuB*::IS*2*; Kan^r; Δ*lacIZ*::EYFP	Resistant	this work
BN1175		W3110; *gyrA*; *btuB*::IS*2*; Kan^r; Δ*lacIZ*::TurboRFP	Resistant	this work
BN1010	pColE2-P9	W3110; *gyrA*	Producer	[14]
BN1083	pColE2-P9	W3110; *gyrA*; Kan^r; Δ*lacIZ*::mCherry	Producer	this work
BN1079	pColE2-P9	W3110; *gyrA*; Kan^r; Δ*lacIZ*::ECFP	Producer	this work
BN1081	pColE2-P9	W3110; *gyrA*; Kan^r; Δ*lacIZ*::EYFP	Producer	this work
BN1173	pColE2-P9	W3110; *gyrA*; Kan^r; Δ*lacIZ*::TurboRFP	Producer	this work
BN1177	pProm-E2	W3110; *gyrA*; Kan^r; Δ*lacIZ*::ECFP	Sensitive	this work
BN1179	pProm-E2	W3110; *gyrA*; Kan^r; Δ*lacIZ*::EYFP	Sensitive	this work
BN1702	pColE2-P9, pProm-E2	W3110; *gyrA*; Kan^r; Δ*lacIZ*::ECFP	Producer	this work
BN1704	pColE2-P9, pProm-E2	W3110; *gyrA*; Kan^r; Δ*lacIZ*::EYFP	Producer	this work
BN1752	pColE2-TT	W3110; *gyrA*; Kan^r; Δ*lacIZ*::EYFP	Producer	this work
BN1703	pColE2-TT, pProm-E2	W3110; *gyrA*; Kan^r; Δ*lacIZ*::ECFP	Producer	this work
BN1705	pColE2-TT, pProm-E2	W3110; *gyrA*; Kan^r; Δ*lacIZ*::EYFP	Producer	this work
BN1799	pColE2-Δ*cel::EYFP*	W3110; *gyrA*; Kan^r; Δ*lacIZ*::ECFP	Producer	this work
**Plasmids**				
pColE2-P9		Colicin E2 plasmid		[14]
pProm-E2	pE2-Crimson derivative	with E2Crimson behind P_{sos}; Amp^r		this work
pColE2-TT	pColE2-P9 derivative	AC to TT mutation in CsrA binding site		this work
pColE2-Δ*cel::EYFP*	pColE2-P9 derivative	in-frame *cel* replacement by *yfp*		this work
