# Supplementary figures and images for: Nutrient-responsive regulation determines biodiversity in a colicin-mediated bacterial community
Source: BMC Biol. 2014 Aug 27;12:68. doi: 10.1186/s12915-014-0068-2 (PMC4161892; doi:10.1186/s12915-014-0068-2)

**A**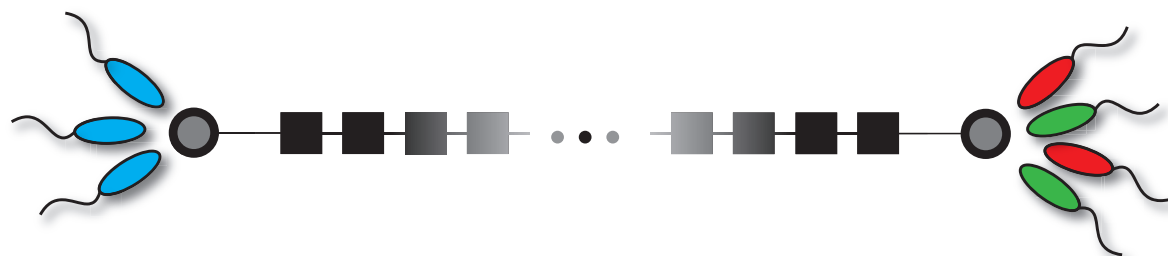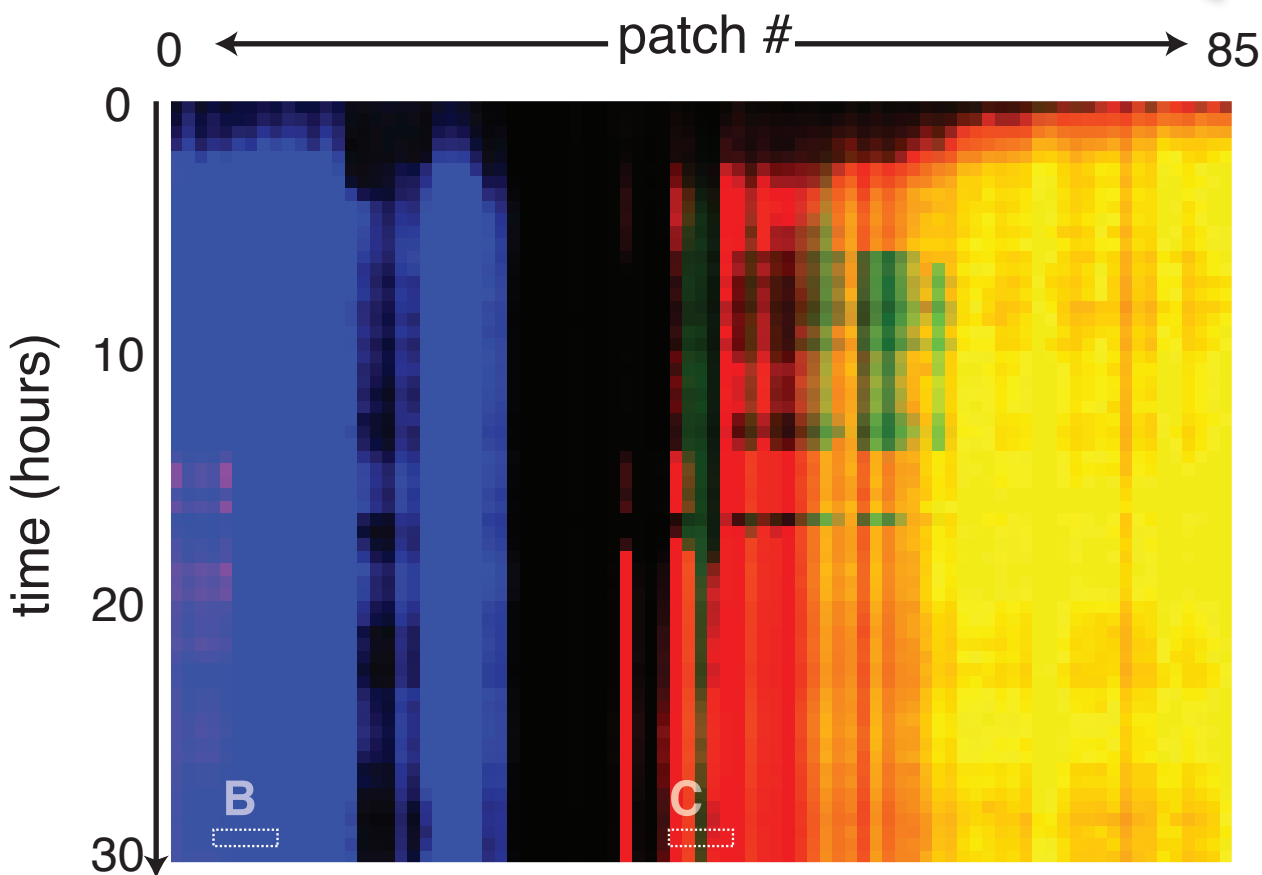**B**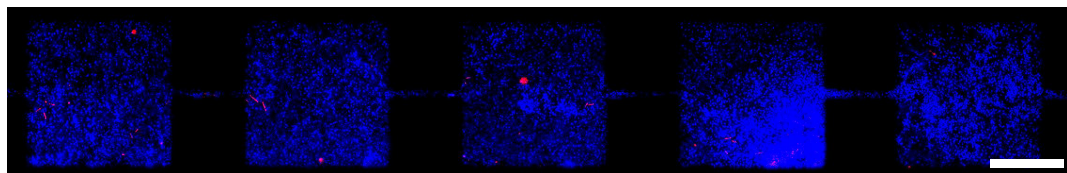**C**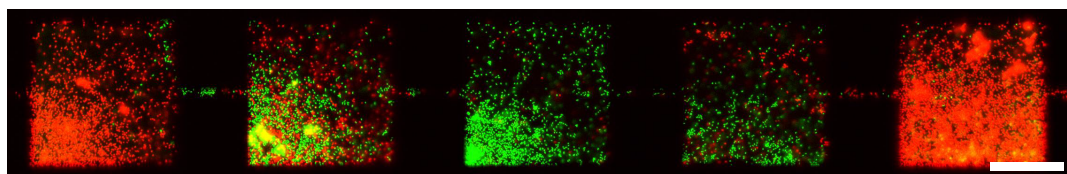

Supplement: Additional file 3 — Figure S2. Asymmetrically inoculated microhabitat. (A) Typical kymograph of a microhabitat asymmetrically inoculated with producer cells from the left and a 50/50 mixture of resistant and sensitive cells from the right. Space is depicted horizontally and time vertically, each pixel represents a single habitat patch and is color-coded according to its producer (blue), resistant (red) and sensitive (green) occupancy. Yellow pixels indicate the presence of resistant and sensitive cells, purple pixels represent patches in which both producer and resistant cells are present, the intensity of the colors scales linearly with the area fraction of the patch that is occupied. Both invading populations readily colonize the habitat, but the population fronts do not collide and an unoccupied zone of several 100 μm separating the populations remains. The separation is not caused by an obstruction, as a few resistant cells can be discerned in the left-most patches colonized by producer cells (purple patches on the left side of the kymograph starting from t=15 hours, and single red cells in (B)). It is also interesting to note that the resistant-sensitive population coming from the right is yellow near the entrance of the habitat but turns red towards the left. This indicates that the population invading from the right is dominated by resistant cells in proximity to the producers and is a mixture of sensitive and resistant cells closer to the entrance. (B,C) Zoom-in of the areas indicated with B and C in the kymograph of (A), scale bars indicate 50 μm. [file 12915_2014_68_MOESM3_ESM.pdf]

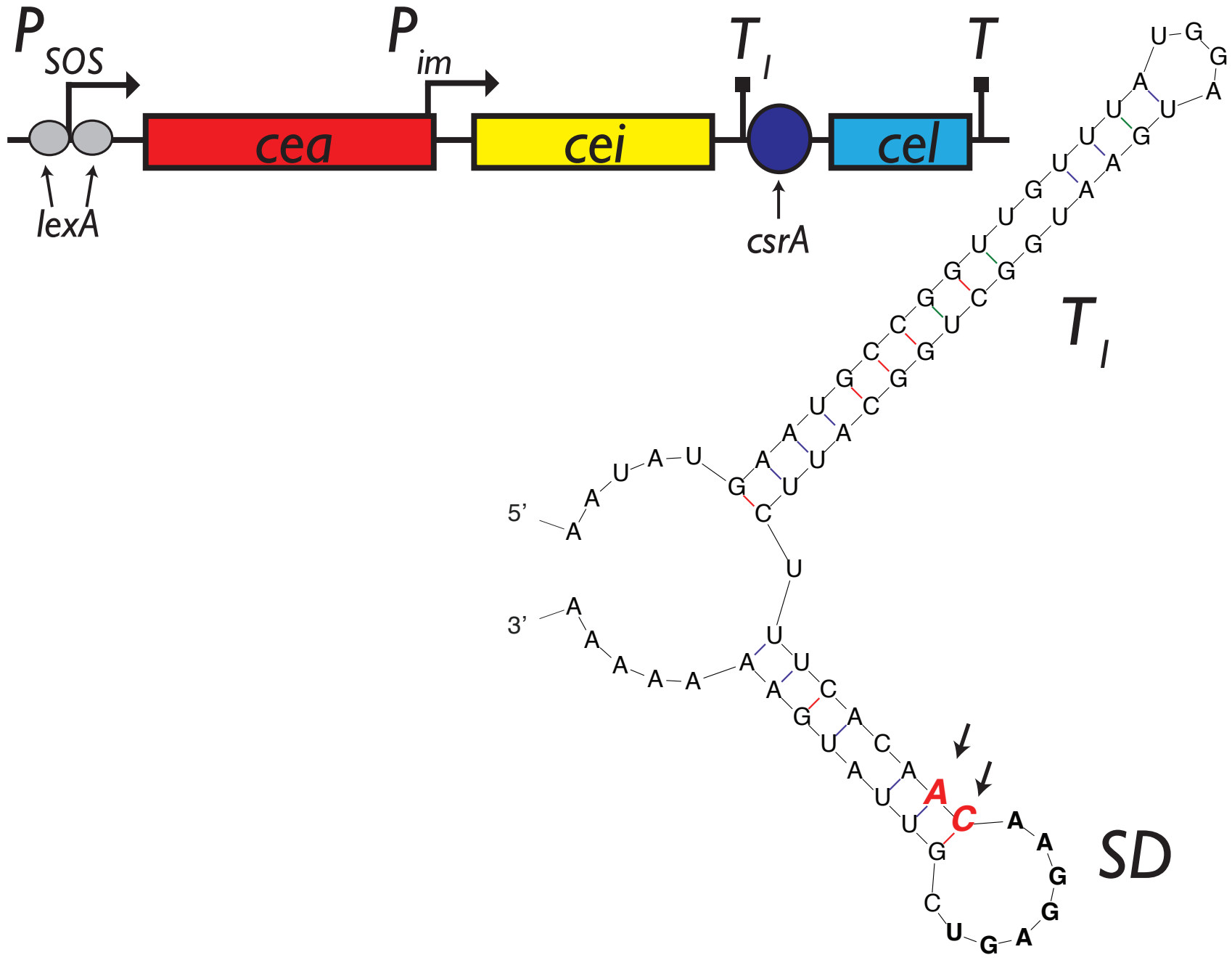

Supplement: Additional file 4 — Figure S3. Organization of the colicin E2 operon. Colicin E2 is a group A nuclease colicin, group A nuclease colicins all have the same general structure and are under control of a LexA repressed SOS promoter (P sos). P sos contains two LexA boxes to which two LexA dimers can bind. When the SOS response is induced, RecA stimulates autocleavage of LexA allowing transcription of the operon. Due to a transcriptional terminator (T 1) between the immunity (cei) and lysis (cel) genes, transcription can result in a full length mRNA and a more abundant shorter mRNA. The full length mRNA corresponds to the entire operon and contains the colicin protein (cea), the immunity protein and the lysis protein; the short mRNA corresponds to the colicin and immunity proteins only. In addition to the SOS promoter, the immunity gene also has its own constitutive promoter (P im) which ensures that there is always enough immunity protein present to bind all colicin and prevent it from killing the producing cell itself. The RNA secondary structure of the T1 terminator as predicted by mfold [61] is depicted on the right. CsrA can bind to this structure and prevent translation of the lysis protein by obscuring the Shine-Dalgarno sequence (SD) and preventing ribosomal binding. Mutating the AC nucleotides (shown in red and indicated by arrows) to TT prevents CsrA binding and thus relieves CsrA-mediated lysis repression. [file 12915_2014_68_MOESM4_ESM.pdf]

**A**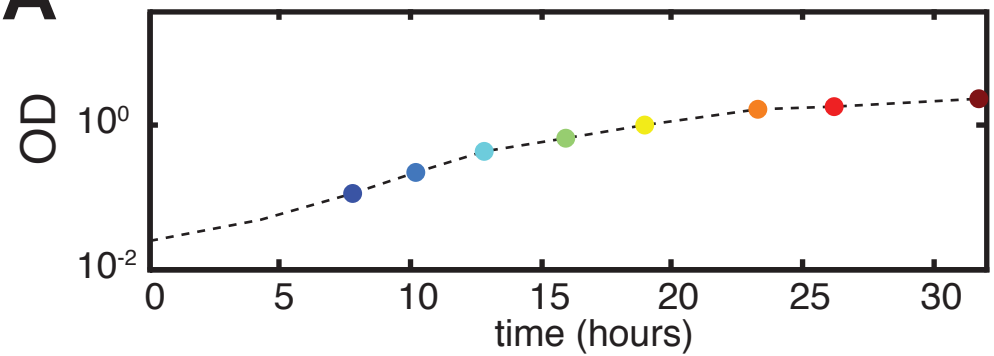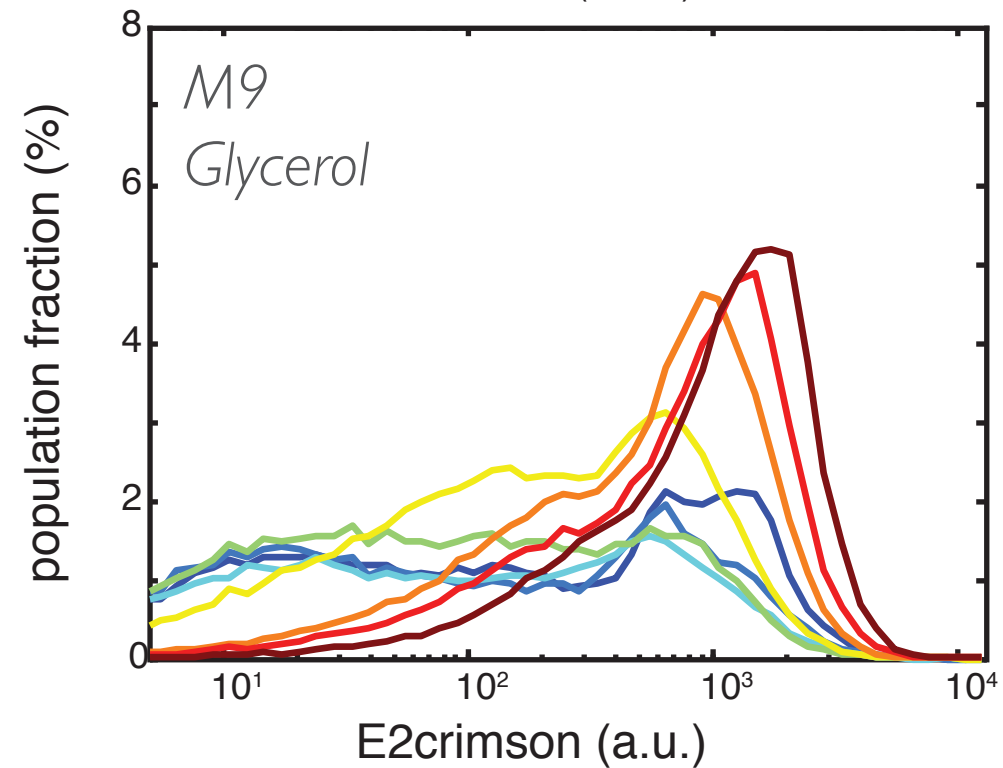**B**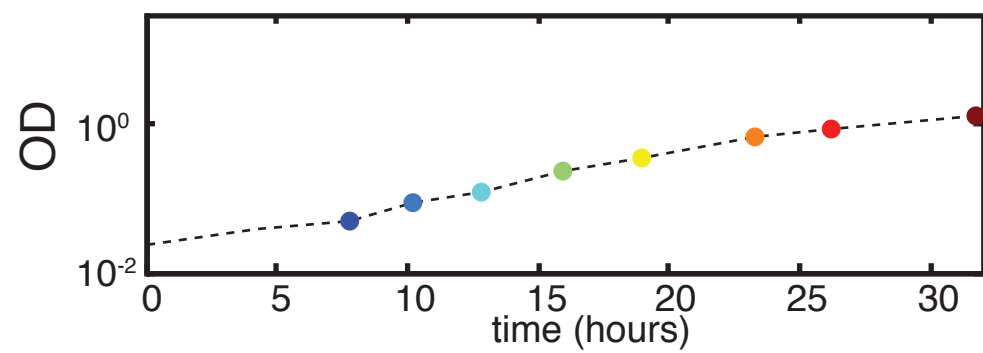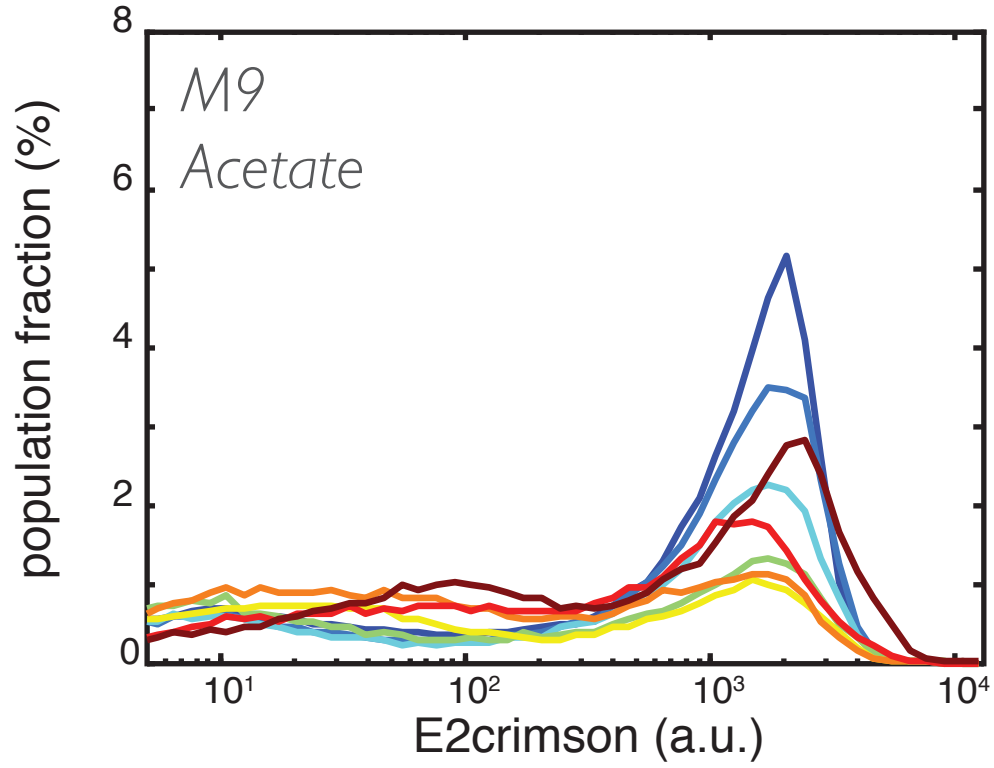

Supplement: Additional file 5 — Figure S4. Expression of the colicin operon in M9-glycerol and M9-acetate. (A,B) Mean histograms of E2crimson expression at various stages during growth of duplicate experiments in M9-glycerol (A) and M9-acetate (B). E2crimson is under control of the colicin E2 promoter and thus serves as a proxy for colicin production. Colors of histograms correspond to time points on the growth curves having the same color, the first bin (cells not expressing E2crimson) is not included for clarity. There is a clear growth phase dependence. Expression profiles are similar to cultures grown in M9-glucose. [file 12915_2014_68_MOESM5_ESM.pdf]
